# Insecticide resistance in *Anopheles arabiensis* from Ethiopia (2012–2016): a nationwide study for insecticide resistance monitoring

**DOI:** 10.1186/s12936-017-2115-2

**Published:** 2017-11-18

**Authors:** Louisa A. Messenger, Josephat Shililu, Seth R. Irish, Gedeon Yohannes Anshebo, Alemayehu Getachew Tesfaye, Yemane Ye-Ebiyo, Sheleme Chibsa, Dereje Dengela, Gunawardena Dissanayake, Estifanos Kebede, Endalew Zemene, Abebe Asale, Mekonnen Yohannes, Hiwot Solomon Taffese, Kristen George, Christen Fornadel, Aklilu Seyoum, Robert A. Wirtz, Delenasaw Yewhalaw

**Affiliations:** 1 Entomology Branch, Centers for Disease Control and Prevention, 1600 Clifton Road, Atlanta, GA 30329-4027 USA; 2 President’s Malaria Initiative Africa Indoor Residual Spraying Project, Abt Associates, Gerji Road, Sami Building, 1st Floor, Addis Ababa, Ethiopia; 3 U.S. Agency for International Development (USAID), Entoto Street, Addis Ababa, Ethiopia; 4 President’s Malaria Initiative Africa Indoor Residual Spraying Project, Abt Associates, 4550 Montgomery Ave., Suite 800 North, Bethesda, MD 20814 USA; 5 Tropical and Infectious Diseases Research Center, Jimma University, Jimma, Ethiopia; 6 Medical and Entomology Unit, Institute of Bio-Medical Sciences, College of Health Sciences, Mekelle University, Mek’ele, Ethiopia; 7 National Malaria Control Programne, Federal Ministry of Health, Addis Ababa, Ethiopia; 8 President’s Malaria Initiative, United States Agency for International Development, Bureau for Global Health, Office of Infectious Disease, 2100 Crystal Drive, Arlington, VA 22202 USA; 9 Department of Medical Laboratory Sciences and Pathology, College of Health Sciences, Jimma University, Jimma, Ethiopia

**Keywords:** Insecticide resistance, *Anopheles arabiensis*, Resistance mechanisms, Intensity assays, Malaria, *kdr*, Ethiopia

## Abstract

**Background:**

Indoor residual spraying (IRS) and long-lasting insecticidal nets (LLINs) remain the cornerstones of malaria vector control. However, the development of insecticide resistance and its implications for operational failure of preventative strategies are of concern. The aim of this study was to characterize insecticide resistance among *Anopheles arabiensis* populations in Ethiopia and describe temporal and spatial patterns of resistance between 2012 and 2016.

**Methods:**

Between 2012 and 2016, resistance status of *An. arabiensis* was assessed annually during the long rainy seasons in study sites from seven of the nine regions in Ethiopia. Insecticide resistance levels were measured with WHO susceptibility tests and CDC bottle bioassays using insecticides from four chemical classes (organochlorines, pyrethroids, organophosphates and carbamates), with minor variations in insecticides tested and assays conducted between years. In selected sites, CDC synergist assays were performed by pre-exposing mosquitoes to piperonyl butoxide (PBO). In 2015 and 2016, mosquitoes from DDT and deltamethrin bioassays were randomly selected, identified to species-level and screened for knockdown resistance (*kdr*) by PCR.

**Results:**

Intense resistance to DDT and pyrethroids was pervasive across Ethiopia, consistent with historic use of DDT for IRS and concomitant increases in insecticide-treated net coverage over the last 15 years. Longitudinal resistance trends to malathion, bendiocarb, propoxur and pirimiphos-methyl corresponded to shifts in the national insecticide policy. By 2016, resistance to the latter two insecticides had emerged, with the potential to jeopardize future long-term effectiveness of vector control activities in these areas. Between 2015 and 2016, the West African (L1014F) *kdr* allele was detected in 74.1% (n = 686/926) of specimens, with frequencies ranging from 31 to 100% and 33 to 100% in survivors from DDT and deltamethrin bioassays, respectively. Restoration of mosquito susceptibility, following pre-exposure to PBO, along with a lack of association between *kdr* allele frequency and *An. arabiensis* mortality rate, both indicate metabolic and target-site mutation mechanisms are contributing to insecticide resistance.

**Conclusions:**

Data generated by this study will strengthen the National Malaria Control Programme’s insecticide resistance management strategy to safeguard continued efficacy of IRS and other malaria control methods in Ethiopia.

**Electronic supplementary material:**

The online version of this article (10.1186/s12936-017-2115-2) contains supplementary material, which is available to authorized users.

## Background

Despite the scaling-up of key diagnostic, treatment and preventative measures, malaria remains a considerable public health problem in Ethiopia, with over 50.6 million (60% of the total population) at significant risk [1]. Transmission of *Plasmodium falciparum* and *Plasmodium vivax* is highly heterogeneous and unstable across the country, concentrated in lowland and highland fringe areas [2]. Unlike other sub-Saharan countries, where malaria morbidity and mortality mainly impacts young children, in Ethiopia, low levels of immunity predispose many individuals to clinically severe malaria and epidemics among all age groups. As part of the National Malaria Strategic Plan (2014–2020), vector control by the National Malaria Control Programme (NMCP), with support from the President’s Malaria Initiative (PMI) and the Global Fund, is based on indoor residual spraying (IRS) and universal coverage campaigns of long-lasting insecticidal nets (LLINs) [1–4].

**Fig. 1:**
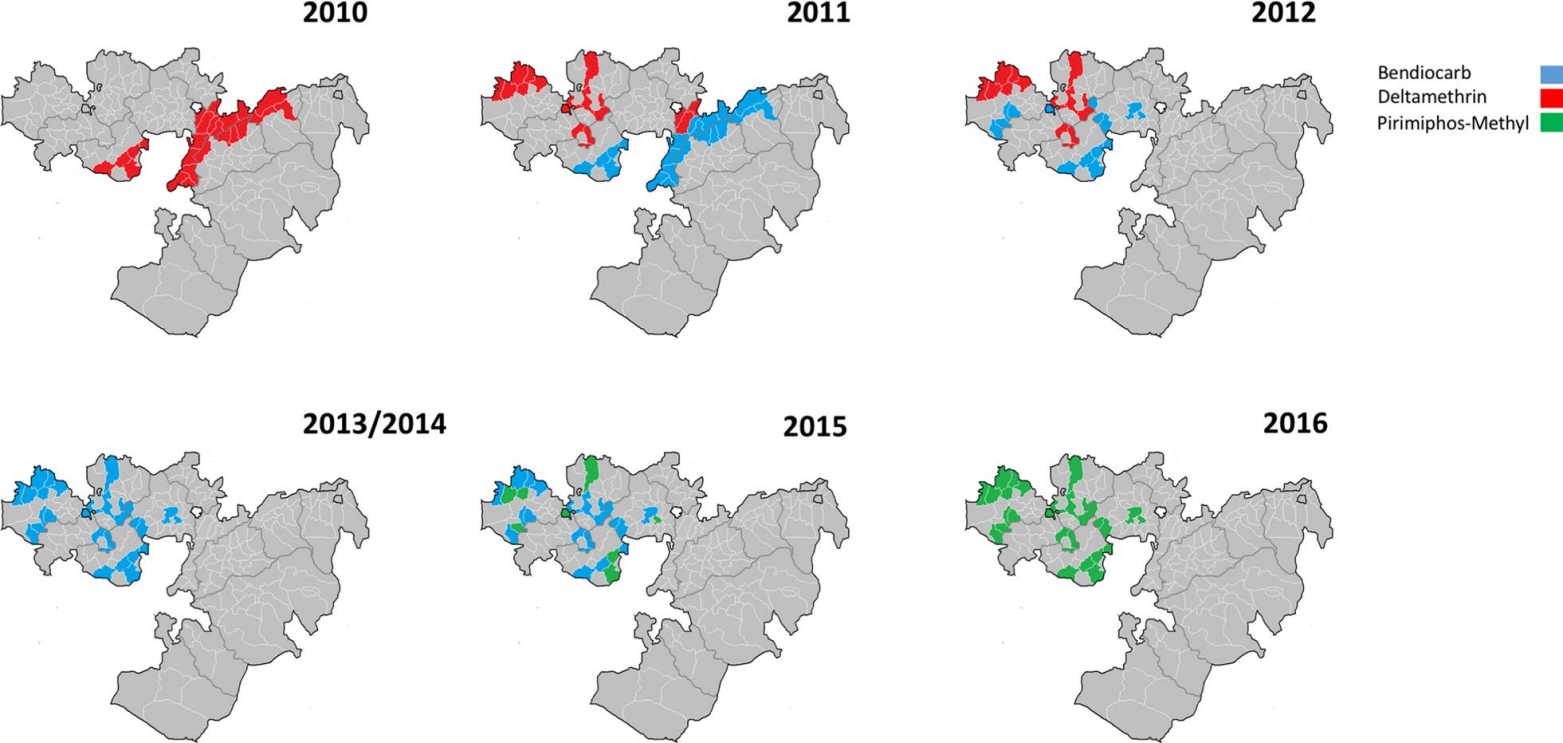
Maps of Oromia Region, Ethiopia displaying insecticides used for IRS activities in PMI-supported districts from 2010 to 2016. Note there have been changes in district boundaries over this time period. Additionally, deltamethrin LLINs have been distributed en masse across all regions since 2010

IRS was first implemented in Ethiopia in 1959 and continues to play a prominent role in malaria control. LLIN coverage has been scaled up since 2005, resulting in over 64 million nets distributed by 2014 [2]. However, the long-term effectiveness of both strategies is currently under threat from widespread emergence of insecticide resistance in the principal malaria vector, *Anopheles arabiensis* [3]. To date, in Ethiopia, *An. arabiensis* has developed resistance against insecticides belonging to all four chemical classes approved for IRS, including DDT (organochlorine), malathion (organophosphate), bendiocarb and propoxur (carbamates) and alpha-cypermethrin, cyfluthrin, deltamethrin, etofenprox, lambda-cyhalothrin and permethrin (pyrethroids) [5–14]. The West African *kdr* (L1014F) mutation has been reported in *An. arabiensis* populations at high frequencies [9, 14, 15] and pre-exposure of *An. arabiensis* to piperonyl butoxide (PBO) significantly increased vector susceptibility to deltamethrin and permethrin [12], suggesting both metabolic and target-site mutation mechanisms are responsible for insecticide resistance. Historically, DDT, and to a lesser extent, malathion were used for IRS in Ethiopia [12]. In 2010, vector control by the NMCP, with support from PMI, discontinued DDT spraying in favour of deltamethrin, which was used initially in combination with bendiocarb from 2011, before being superseded exclusively by bendiocarb and propoxur in 2013. In 2015, in response to incipient resistance, PMI-supported IRS activities were based on bendiocarb in 28 districts (and focal pirimiphos-methyl application in 8 districts) and in 2016, pirimiphos-methyl replaced bendiocarb in all PMI-supported districts [2] (Fig. 1 and Additional file 1: Table S1).

Considering only a limited number of alternate insecticides are available for public health use, the aim of this study was to characterize contemporary nationwide insecticide resistance in *An. arabiensis* populations and describe longitudinal trends in resistance between 2012 and 2016, to strengthen the NMCP’s insecticide resistance monitoring and management strategy and safeguard continued efficacy of IRS in Ethiopia [16].

## Methods

### Study sites

Data are from study sites in seven of the nine regions [Afar, Amhara, Benishangul Gumuz, Gambela, Oromia, Southern Nations, Nationalities, and Peoples’ Region (SNNPR), and Tigray] in Ethiopia between 2012 and 2016.

### Susceptibility tests

For all susceptibility tests, mosquito larvae were collected by dipping from a range of breeding sites in each study area and reared to adults under standard insectary conditions (temperature 25 ± 2 °C, relative humidity 80 ± 10%). Bioassays were conducted annually during the long rainy season (June–September). WHO tube tests and CDC bottle bioassays were used to determine susceptibility levels of *Anopheles gambiae* s.l. populations (henceforth *An. arabiensis*) to different insecticides, with minor variations in insecticides tested and assays conducted between years (Additional file 1: Table S2). From 2014 onwards, CDC resistance intensity assays were undertaken and synergist assays were also performed by pre-exposing mosquitoes to PBO in selected sites. For all assays, care was taken during storage and field transportation of insecticide-impregnated papers and technical grade insecticide stock solutions to reduce heat exposure and minimize potential loss of efficacy.

### WHO susceptibility tests

World Health Organization (WHO) susceptibility tests for the following eleven insecticides with diagnostic doses (alpha-cypermethrin (0.05%), bendiocarb (0.1%), DDT (4%), deltamethrin (0.05%), etofenprox (0.5%), fenitrothion (1%), lambda-cyhalothrin (0.05%), malathion (5%), permethrin (0.75%), pirimiphos-methyl (0.25%) and propoxur (0.1%)) were conducted according to WHO guidelines [17]. The diagnostic dose of insecticides used on papers is generally twice the LC_99_ values systematically determined from baseline studies in multiple locations [18]. In 2012, four replicates of 20–25 non-blood fed, 2-3 days old adult female mosquitoes were exposed to different insecticide-impregnated papers in WHO tubes for 1 h (except in the case of fenitrothion where mosquitoes were exposed for 2 h) and in parallel, one replicate of control mosquitoes (20–25 mosquitoes per tube) was exposed to oil-impregnated papers; from 2013 onwards, two control replicates using 25 mosquitoes were performed. For all assays, mosquito mortality was recorded after a 24-h holding period. Cotton wool soaked in 10% sugar solution was provided to mosquitoes on top of the holding tube and optimum temperature and relative humidity was maintained using a damp towel placed on top of boxes where holding tubes were kept.

### CDC bottle bioassays

CDC bottle bioassays for seven insecticides (alpha-cypermethrin, bendiocarb, DDT, deltamethrin, lambda-cyhalothrin, permethrin and propoxur) were conducted according to published guidelines [19]. Stock solutions were prepared by diluting technical grade insecticide in 50 mL of acetone. Each Wheaton 250 mL bottle along with its cap was coated with 1 mL of stock solution (12.5 µg/bottle for alpha-cypermethrin, bendiocarb, deltamethrin, lambda-cyhalothrin and propoxur, 100 µg/bottle for DDT and 21.5 µg/bottle for permethrin) by rolling and inverting the bottles. In each test, a control bottle was coated with 1 mL of acetone. Following coating, bottles were covered with mats and left to dry. Approximately, 10–25 non-blood fed, 2–3 days old adult female mosquitoes were introduced into each bottle using a mouth aspirator and mortality was recorded at 15 min intervals up to 30 min for all insecticides except DDT; for this assay mosquitoes were exposed for 45 min. From 2014 onwards, CDC resistance intensity assays were performed by testing 1, 2, 5 and 10 times the concentration required to kill all mosquitoes (LC_100_), as determined in a series of baseline experiments [19], and in selected sites, synergist assays were also conducted by pre-exposing mosquitoes to PBO for 1 h (100 µg/bottle).

### Molecular identification of *Anopheles gambiae* species complex

Mosquitoes used in bioassay tests were identified morphologically using standard keys [20]. In 2015 and 2016, sub-samples of both surviving and dead mosquitoes from WHO tests were randomly selected by insecticide (DDT and deltamethrin), site and region for molecular species identification and *kdr* allele detection at the Molecular Biology Laboratory, Tropical and Infectious Diseases Research Centre (TIDRC) of Jimma University. Genomic DNA was extracted following the procedure described by Collins et al. [21]. DNA was re-suspended in 25 ml sterile TE-buffer (10 mM Tris–HCl pH 8, 1 mM EDTA). Molecular identification of *An. gambiae* s.l. was carried out by species-specific PCR using primers for *An. gambiae* s.s., *An. arabiensis* and *Anopheles quadriannulatus* species B (*Anopheles amharicus*) according to Scott et al. [22], with modifications [9, 23]. Briefly, genomic DNA was mixed with primers AR (5′-AAGTGTCCTTCTCCATCCRA-3′; specific for *An*. *arabiensis*), AG (5′-CTGGTTTGGTCGGCACGTTT-3; specific for *An. gambiae* s.s.), QD-b (5′-AGTGTCCAATGTCTGTGAAG-3′; specific for *An. quadriannulatus* species B) and UN (5′-GTGTGCCCCTTCCTCGATGT-3′; common for all species) in a 25 μL reaction. Amplification reactions contained 1 μL of DNA, 1.5 mM MgCl_2_, 10 mM Tris–HCl (pH 8.4), 50 mM KCl, 0.1% Triton X-100, 200 μM of dNTPs (Amersham, Buckinghamshire, United Kingdom), 25 pmol of primers AR, AG, QD-b and UN and 0.25 U of SilverStar DNA polymerase (Eurogentec, Seraing, Belgium) [24]. PCR reaction conditions are described in Scott et al. [22]. Amplified PCR products were visualized on 2% agarose gels, stained with ethidium bromide. *An. arabiensis* strain from the Sekoru colony, maintained at the Vector Biology and Control Research Unit, TIDRC, Jimma University, was used as a positive control.

### Detection of resistance mutations

West African *kdr* (L1014S) and East African *kdr* (L1014F) alleles were detected using adapted protocols [24] for allele-specific PCR (AS-PCR), developed by Martinez-Torres et al. [25] and Ranson et al. [26]. Primers Agd1 (5′-ATAGATTCCCCGACCATG-3′), Agd2 (5′-AGACAAGGATGATGAACC-3′), Agd3 (5′-AATTTGCATTACTTACGACA-3′) and Agd4 (5′-CTGTAGTGATAGGAAATTTA-3′) were used to detect the L1014F allele (AS-PCR Agd3), whereas primers Agd1, Agd2, Agd4 and Agd5 (5′-TTTGCATTACTTACGACTG-3′) were used to detect the L1014S allele (AS-PCR Agd5). Amplifications were performed in 50 μL reactions containing 2 μL DNA, 1× Qiagen PCR buffer, 0.5 mM MgCl_2_, 100 nM of each primer, 200 μM dNTPs, and 1U *Taq* DNA polymerase (*Taq* PCR core kit, Qiagen, Hilden, Germany). The cycling conditions were: initial 94 °C denaturation for 5 min, 10 cycles of 1 min denaturation at 94 °C, 30 s annealing at 54 °C and 30 s extension at 72 °C, followed by 30 cycles of 1 min denaturation at 94 °C, 30 s annealing at 47 °C and 30 s extension at 72 °C, and a final extension at 72 °C for 10 min. Amplification products were visualized on 2% agarose gels, stained with ethidium bromide. *An. arabiensis* from the Sekoru colony (a *kdr* negative mosquito strain) was used as a negative control.

### Data analysis

Data were interpreted according to the WHO guidelines [17]; mortality of 98% or higher in susceptibility tests indicates susceptibility, mortality of 90–97% is suggestive of resistance and mortality of less than 90% indicates resistance. Mortality was corrected using Abbott’s formula, when mortality in control assays was between 5 and 20% [17, 27]. A bioassay was repeated if control mortality exceeded 20%. Per site, mean percent mosquito mortality was determined across all replicates for a given insecticide. Pearson’s Chi squared tests were used to evaluate the association of *kdr* frequency with WHO assay results and to test for deviations from Hardy–Weinberg equilibrium. Cohen’s Kappa (κ) was calculated to quantify the magnitude of agreement between WHO susceptibility tests and CDC bottle bioassays [28]; values were interpreted as poor (κ ≤ 0), slight (0 < κ ≤ 0.2), fair (0.2 < κ ≤0.4), moderate (0.4 < κ ≤0.6), substantial (0.6 < κ ≤0.8) and almost perfect agreement (0.8 < κ ≤1.0) [29]. All statistical analyses were performed in Stata/IC 14.2 (Stata Corp., College Station, USA) with the level of significance set at α = 0.05.

## Results

### WHO susceptibility tests

**Fig. 2:**
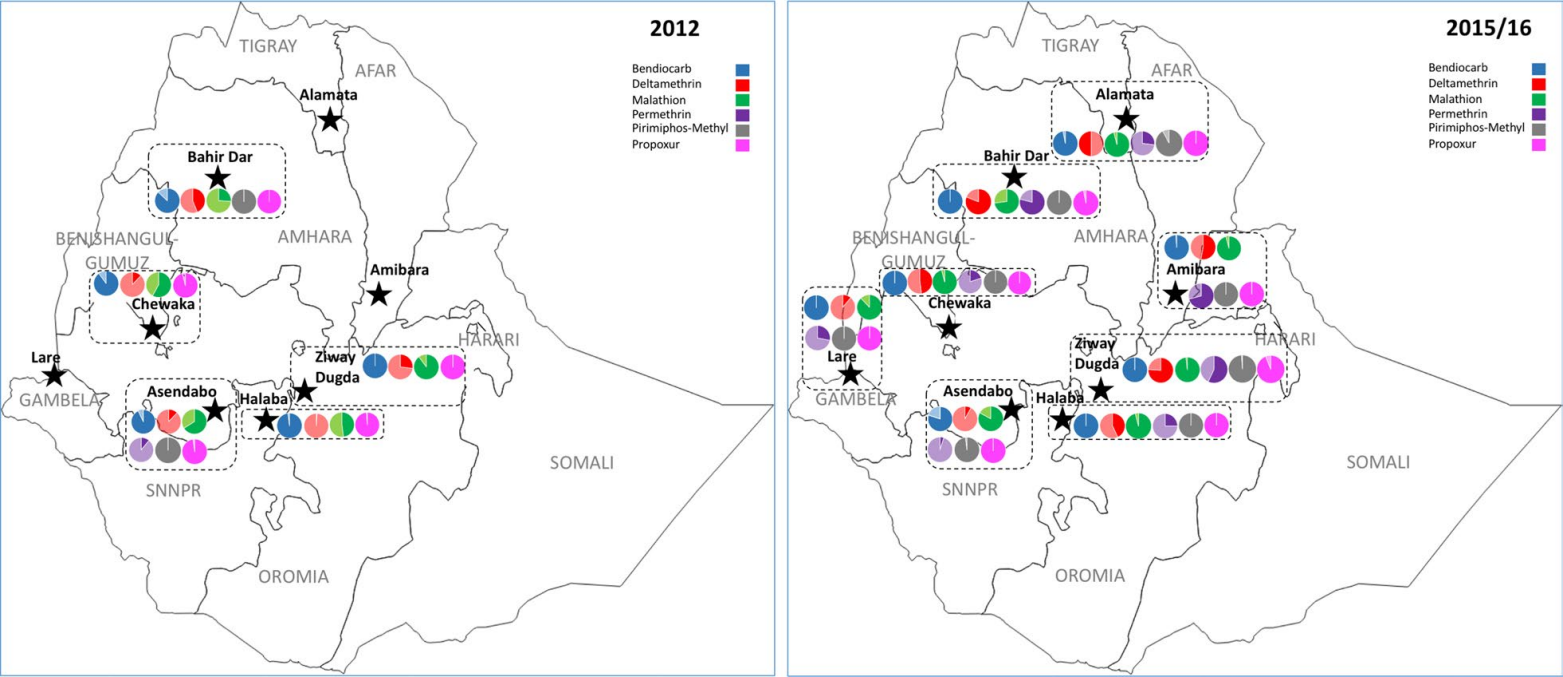
Maps of Ethiopia displaying trends in *An. arabiensis* susceptibility levels to bendiocarb, deltamethrin, malathion, permethrin, pirimiphos-methyl and propoxur, as measured using WHO susceptibility tests, between 2012 (left) and 2015/16 (right). Vector populations were sampled from eight sentinel sites (Alamata, Amibara, Asendabo, Bahir Dar, Chewaka, Halaba, Lare and Ziway Dugda) in six regions. Legend colours indicate the insecticide under evaluation, with darker shading denoting average mosquito mortality

Results from WHO susceptibility tests conducted in eight sentinel sites (Alamata, Amibara, Asendabo, Bahir Dar, Chewaka, Halaba, Lare and Ziway-Dugda) between 2012 and 2016 are summarized in Table 1 and Fig. 2. WHO tests conducted in additional areas from 2013 onwards are detailed in Additional file 1: Tables S3–S6 by year.

**Table 1:**
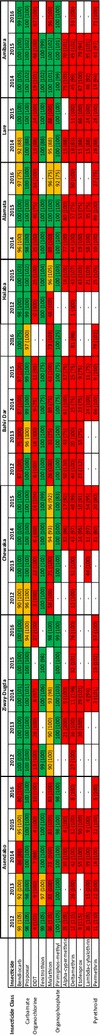
Percentage corrected mortality (and numbers tested)of *Anopheles arabiensis* in WHO susceptibility tests conductedin eight national sentinel sites in Ethiopia 2012–2016

In 2012, high levels of pyrethroid resistance were evident across Ethiopia, with *An. arabiensis* mortality levels of 50% or less for alpha-cypermethrin, deltamethrin, etofenprox, lambda-cyhalothrin and permethrin. Similarly, all mosquito populations were highly resistant to DDT (mortality ranging from 0 to 13%) and demonstrated variable levels of susceptibility to the organophosphate malathion (26–90% mortality) (Table 1). In contrast, *An. arabiensis* was fully susceptible to organophosphates fenitrothion and pirimiphos-methyl and the carbamate propoxur, with the exception of Chewaka, where average mortality for the latter was 96%. Low levels of developing bendiocarb (carbamate) resistance were detected in three study sites, Asendabo, Bahir Dar and Chewaka (mortality of 93, 87 and 90%, respectively).

In 2015–2016, most *An. arabiensis* populations presented consistent susceptibility profiles, with minor variations in some resistance levels; notably in four sites (Asendabo, Chewaka, Halaba and Ziway-Dugda) susceptibility to malathion increased over time (from 66 to 83%, 58 to 96%, 48 to 96% and 90 to 98% average mosquito mortality, respectively), while mosquito mortality to pirimiphos-methyl began to decline slightly in Asendabo and Ziway-Dugda (from 100 to 98% and 100 to 99% mortality, respectively) and possible resistance to propoxur emerged in Ziway-Dugda (94% mortality) and Bahir Dar (97% mortality). Resistance to pirimiphos-methyl and propoxur was also detected in additional study sites from Oromia region; *An. arabiensis* mortality was 85% in Babile in 2016 to pirimiphos-methyl, and 75% and 95% to propoxur in Abaya in 2013 and in Nono in 2016, respectively (Additional file 1: Tables S3, S6). Interestingly, vectors from an area of sesame cultivation sampled in 2016 (Metema; Amhara Region) demonstrated almost complete susceptibility to deltamethrin (average mortality of 99%; Additional file 1: Table S6).

Similar longitudinal trends were observed in Alamata, Lare and Amibara, where resistance monitoring began in 2014 (Table 1). In these areas, at baseline, vector populations were also resistant to pyrethroids and DDT, fully susceptible to fenitrothion, pirimiphos-methyl and propoxur and potentially resistant to bendiocarb in Alamata and Lare (mortality of 96 and 92%, respectively). In these latter two sites, resistance to malathion was also detected (mortality of 89 and 95%, respectively). By 2016, putative resistance to pirimiphos-methyl had developed in Alamata (mortality of 92%) and to malathion in Amibara (mortality of 96%). On a yearly basis, levels of resistance fluctuated within sites, in some cases modestly (e.g. in Asendabo, mortality to bendiocarb oscillated from 93% in 2012, to 86% in 2014, 95% in 2015 and 80% in 2016) and in others more dramatically, beyond what might be expected of stochastic variation (e.g. in Bahir Dar, mortality to malathion ranged from 26% in 2012, to 89% in 2014 and 43% in 2015).

### CDC bottle bioassays

**Fig. 3:**
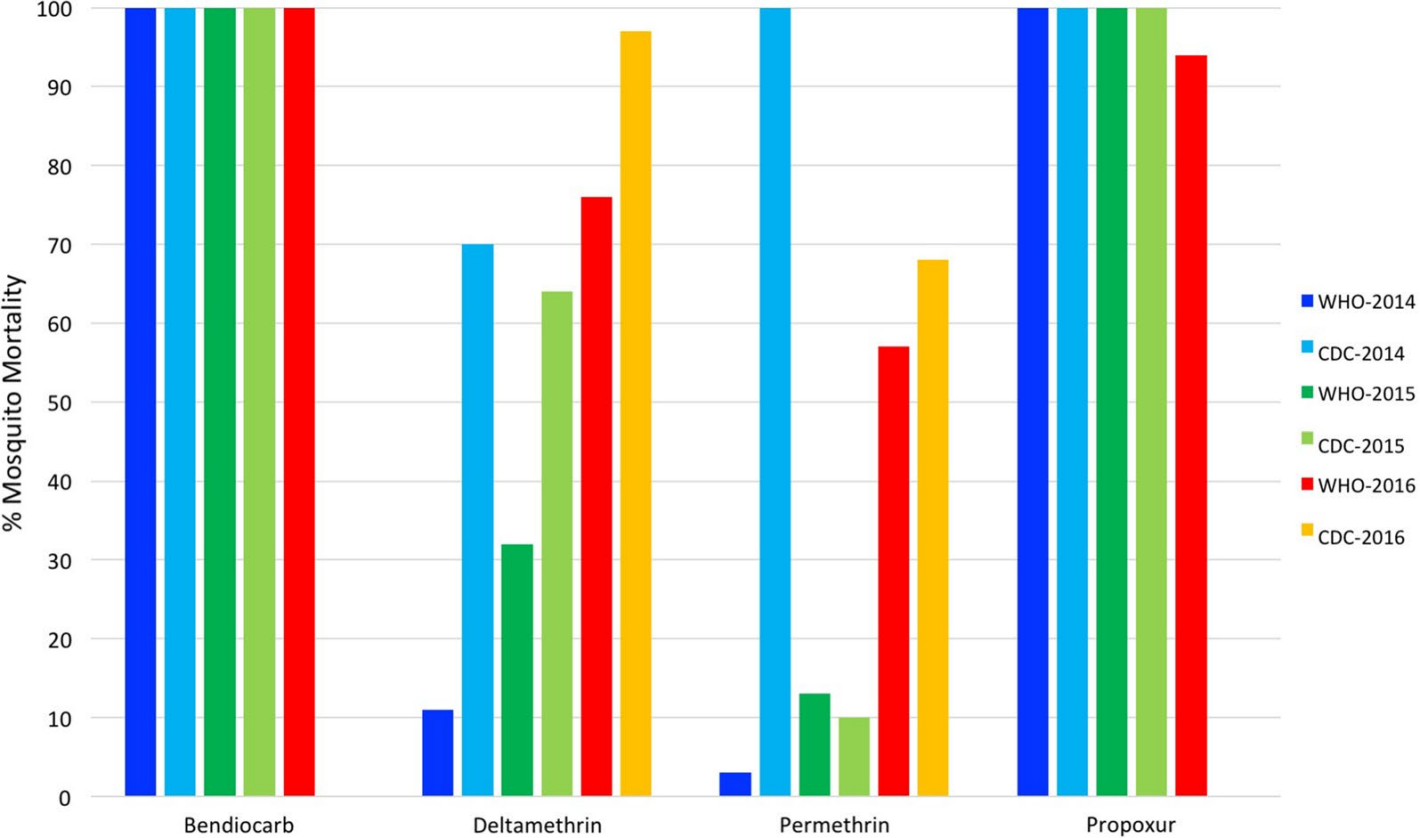
Comparison of WHO and CDC bottle bioassays (at both respective diagnostic doses), conducted using bendiocarb, deltamethrin, permethrin and propoxur in Ziway-Dugda from 2014 to 2016

From 2013 onwards, CDC bottle bioassays, resistance intensity and synergist assays were conducted in additional study sites, which differed between years (Additional file 1: Tables S7–S10). Data from Ziway-Dugda, where these tests were performed routinely throughout the monitoring period are presented in Fig. 3 and Additional file 1: Table S11, in comparison with concurrent WHO bioassays. Results from both WHO tests (diagnostic dose) and CDC bottle bioassays (2X) were concordant for bendiocarb and propoxur; vector populations were fully susceptible to both insecticides until 2016. However, levels of pyrethroid resistance were not comparable between techniques, e.g. *An. arabiensis* mortality in 2014 to deltamethrin was 11% in WHO tests, compared to 70% in CDC bottle bioassays at the same discriminatory dose (κ ≤ 0, for all comparisons between pyrethroid assays in 2014, 2015 and 2016).

While consistently high levels of pyrethroid resistance were observed using WHO tests, examination of CDC bioassays from additional study areas identified some completely susceptible *An. arabiensis* populations, e.g. mortality was 100% to deltamethrin in Abedogoro at the equivalent WHO diagnostic dose (2X) in 2015. Focal patterns of pyrethroid resistance were also apparent in 2013, where vector populations, susceptible to one or more pyrethroids, were identified in eleven areas across three regions (Additional file 1: Table S7). In these sites resistance to a particular pyrethroid was not necessarily associated with increased tolerance to another, e.g. in Gobu-Seyo (Oromia Region) mosquito mortality was 76, 49, 98 and 8% to alpha-cypermethrin, deltamethrin, lambda-cyhalothrin and permethrin at the CDC  diagnostic doses, respectively. In other areas with high levels of pyrethroid resistance, CDC resistance intensity assays detected some mosquitoes capable of surviving ten times the diagnostic dose of deltamethrin or permethrin (e.g. average mortality of 75 and 65% in Asendabo in 2015, respectively; Additional file 1: Table S9). High intensities of DDT resistance were also observed in these areas, e.g. 95% of mosquitoes survived ten times the diagnostic dose in Wondogenet in 2014 (Additional file 1: Table S8).

In all sites where mosquitoes were pre-exposed to PBO, a synergist that interferes with oxidase activity, resistance to both deltamethrin and permethrin was reduced, increasing mortality to 87–100% when mosquitoes were exposed to the 1X dose (Additional file 1: Tables S8–S11).

### Molecular detection of species and resistance mutations

Following WHO susceptibility tests in 2015, three hundred and sixty-four surviving and dead *An. gambiae* s.l. specimens were randomly selected from eight study sites in six regions [Asendabo, Chewaka, Ziway-Dugda (Oromia region), Bahir Dar (Amhara region), Amibara (Afar region), Alamata (Tigray region), Halaba (SNNPR) and Abobo (Gambela region)] and were assayed to determine species. Of these, 94.8% (345/364) of samples amplified and were identified as *An. arabiensis*. Non-amplifiers were evenly distributed across all study sites, except Amibara and Ziway-Dugda where all specimens were successfully classified. In 2016, six hundred and sixty-two *An. gambiae* s.l. were collected from ten study sites in six regions: Asendabo, Babile, Nono and Ziway-Dugda (Oromia region), Metema (Amhara region), Amibara (Afar region), Abobo (Gambela region), Alamata and Humera (Tigray region) and Arba Minch (SNNPR), and 86.7% (574/662) of specimens were confirmed as *An. arabiensis.* Higher proportions of non-amplifiers were collected from Metema (17.6% of total mosquitoes sampled), Nono (30%), Arba Minch and Abobo (both 17.5%), than other neighboring areas (range of remaining sites: 5–12.5%).

In 2015, the presence of *kdr* was also assessed in matched mosquito samples (Table 2). The West African (L1014F) *kdr* allele was identified in 75.5% (n = 275/364) of specimens (215 alive and 60 dead), with allele frequencies ranging from 50 to 100% and 13 to 88% in surviving and dead *An. arabiensis* from DDT bioassays, respectively, and from 36 to 100% and 13 to 100% in surviving and dead *An. arabiensis* from deltamethrin bioassays, respectively. The majority of vectors surviving bioassays were homozygous for *kdr* (54.0%; 116/215) compared to those that died (23.3%; 14/60); 29.3% (63/215) and 36.7% (22/60) were heterozygous for *kdr*, respectively. The *kdr*-West allele was present in 100% of surviving vectors from one study site (Alamata; Tigray region) and 94% in another (Halaba; the SNNPR). Deviations from Hardy–Weinberg equilibrium were only observed in *An. arabiensis* survivors of deltamethrin bioassays in Asendabo and Lare (χ^2^ = 7.13; *p* = 0.0076 and χ^2^ = 7.75; *p* = 0.0054 respectively) (Table 2). There was no significant association between *kdr* allele frequency and *An. arabiensis* mortality following exposure to DDT or deltamethrin (*p* = 0.227 and *p* = 0.208, respectively). The East African (L1014S) *kdr* allele was not detected in any samples assayed.

**Table 2:** Genotypic and *kdr* allele frequencies in *Anopheles arabiensis* from eight national sentinel sites in Ethiopia, 2015

Region	Village/site	Insecticide	Survival status after exposure	# mosquitoes tested	Homozygote mutation (RR)	Heterozygote mutation (RS)	Homozygote wild type (SS)	*Kdr* allele frequency		*χ* ^2^ test	*p* value	% mortality in WHO tests	Resistance status
								*R*	*S*				
Oromia	Chewaka	DDT	Alive	15	1	6	6	0.31	0.69	0.345	0.557	14	R
			Dead	5	1	1	3	0.3	0.7	1.372	0.242		
		Deltamethrin	Alive	15	2	4	5	0.36	0.64	1.441	0.23	48	R
			Dead	5	1	1	0	0.75	0.25	1.889	0.169		
	Asendabo	DDT	Alive	30	14	6	0	0.85	0.15	3.749	0.0528	4	R
			Dead	4	0	1	3	0.13	0.88	0.0892	0.765		
		Deltamethrin	Alive	30	16	4	4	0.75	0.25	7.126	0.0076	32	R
			Dead	6	2	2	1	0.6	0.4	0.282	0.595		
	Ziway-Dugda	DDT	Alive	15	3	4	3	0.5	0.5	1.988	0.164	–	–
			Dead	–	–	–	–	–	–	–	–		
		Deltamethrin	Alive	15	3	4	3	0.5	0.5	1.988	0.164	32	R
			Dead	10	0	3	5	0.19	0.81	0.734	0.391		
Amhara	Bahir Dar	DDT	Alive	15	3	4	1	0.63	0.37	3.287	0.0698	11	R
			Dead	5	0	1	3	0.13	0.87	0.262	0.609		
		Deltamethrin	Alive	15	3	6	3	0.5	0.5	0.6	0.439	25	R
			Dead	5	0	1	3	0.13	0.87	0.262	0.609		
SNNPR	Halaba	DDT	Alive	15	8	1	0	0.94	0.06	2.42	0.12	25	R
			Dead	5	1	0	2	0.3	0.7	2.855	0.0911		
		Deltamethrin	Alive	15	5	2	1	0.75	0.25	3.741	0.0531	43	R
			Dead	5	2	1	1	0.63	0.37	0.906	0.341		
Tigray	Alamata	DDT	Alive	9	9	0	0	1	0	–	–	40	R
			Dead	5	3	1	0	0.88	0.12	0.271	0.602		
		Deltamethrin	Alive	8	6	0	0	1	0	–	–	57	R
			Dead	3	1	0	0	1	0	–	–		
Afar	Amibara	DDT	Alive	14	6	2	1	0.78	0.22	2.535	0.111	48	R
			Dead	5	1	2	2	0.4	0.6	0.139	0.709		
		Deltamethrin	Alive	15	2	7	3	0.46	0.54	0.893	0.345	49	R
			Dead	5	1	3	1	0.5	0.5	0.2	0.655		
Gambela	Lare	DDT	Alive	30	17	10	3	0.74	0.26	0.733	0.392	24	R
			Dead	5	0	4	0	0.5	0.5	3.4	0.0652		
		Deltamethrin	Alive	30	18	3	3	0.81	0.19	7.746	0.00538	11	R
			Dead	5	1	1	0	0.75	0.25	1.889	0.169		

In 2016, all mosquito specimens confirmed as *An. arabiensis* (n = 562; with the exception of 8 and 4 samples from Metema and Alamata, respectively) were assayed for *kdr* (Table 3). L1014F *kdr* was detected in 73.5% (n = 413/562) of *An. arabiensis* (322 alive and 91 dead), with allele frequencies ranging from 31 to 89% and 10 to 100% in surviving and dead mosquitoes from DDT bioassays, respectively, and from 33 to 100% and 6 to 94% in surviving and dead mosquitoes from deltamethrin bioassays, respectively. There was no significant differences in *kdr* allele frequency between years among DDT or deltamethrin bioassay survivors (*p* = 0.229 and *p* = 0.158, respectively). As previously, the majority of *An. arabiensis* surviving bioassays were homozygous for *kdr* (42.2%; 136/322) compared to those that died (28.6%; 26/91); 33.2% (107/322) and 28.6% (26/91) were heterozygous for *kdr*, respectively. The *kdr*-West allele was fixed in surviving vectors from two study sites (Babile and Nono; Oromia region); in Alamata, *kdr* frequencies had declined from 100% in 2015 to 62% and 57% among survivors of DDT and deltamethrin bioassays, respectively. Consistent with results from 2015, deviations from Hardy–Weinberg equilibrium were identified among *An. arabiensis* in Asendabo (χ^2^ = 9.71; *p* = 0.0018 for deltamethrin survivors) (Table 3) and in additional vector populations in Abobo (χ^2^ = 13.60; *p* = 0.00023, χ^2^ = 3.83; *p* = 0.05 for survivors of DDT and deltamethrin bioassays, respectively), Arba Minch (χ^2^ = 4.87; *p* = 0.027 for DDT survivors), Alamata (χ^2^ = 4.83; *p* = 0.028 for deltamethrin survivors) and Amibara (χ^2^ = 5.62; *p* = 0.018 and χ^2^ = 6.53; *p* = 0.011 for DDT and deltamethrin survivors, respectively). There was no significant association between *kdr* allele frequency and *An. arabiensis* mortality following exposure to DDT or deltamethrin (*p* = 0.231 and *p* = 0.267, respectively).

**Table 3:** Genotypic and *kdr* allele frequencies in *Anopheles arabiensis* from ten national sentinel sites in Ethiopia, 2016

Region	Site	Insecticide	Survival status after exposure	# mosquitoes tested	Homozygote mutation (RR)	Heterozygote mutation (RS)	Homozygote wild type (SS)	*kdr* allele frequency		*χ* ^2^ test	*P* value	% mortality in WHO tests	Resistance status
								R	S				
Oromia	Babile	DDT	Alive	13	7	2	0	0.89	0.11	1.33	0.249	11	R
			Dead	9	9	0	0	1	0	–	–		
		Deltamethrin	Alive	15	14	0	0	1	0	–	–	22	R
			Dead	9	8	1	0	0.94	0.167	1.431	0.232		
	Nono	DDT	Alive	12	2	3	3	0.44	0.56	1.635	0.201	29	R
			Dead	9	0	3	3	0.25	0.75	1.444	0.229		
		Deltamethrin	Alive	8	7	0	0	1	0	–	–	52	R
			Dead	6	2	2	2	0.5	0.5	0.667	0.414		
	Asendabo	DDT	Alive	28	9	11	6	0.56	0.44	0.635	0.426	10	R
			Dead	0	0	0	0	0	0	–	–		
		Deltamethrin	Alive	25	3	12	6	0.43	0.57	9.706	0.00184	8	R
			Dead	7	1	1	3	0.3	0.7	1.551	0.213		
	Ziway-Dugda	DDT	Alive	30	9	10	11	0.47	0.53	3.272	0.0705	27	R
			Dead	10	0	2	8	0.1	0.9	0.123	0.725		
		Deltamethrin	Alive	9	2	4	3	0.44	0.56	0.092	0.762	76	R
			Dead	8	0	1	7	0.06	0.94	0.04	0.841		
Amhara	Metema	DDT	Alive	11	1	3	4	0.31	0.69	0.914	0.339	74	R
			Dead	8	0	1	4	0.2	0.8	1.516	0.218		
		Deltamethrin	Alive	1	1	0	0	1	0	–	–	99	S
			Dead	0	0	0	0	0	0	–	–		
SNNPR	Arba Minch	DDT	Alive	28	12	7	7	0.6	0.4	4.869	0.0273	51	R
			Dead	10	1	3	4	0.31	0.69	0.505	0.477		
		Deltamethrin	Alive	22	12	8	2	0.73	0.27	0.157	0.692	41	R
			Dead	6	2	1	3	0.41	0.59	2.619	0.106		
Tigray	Alamata	DDT	Alive	30	10	12	4	0.62	0.38	0.552	0.458	34	R
			Dead	9	0	4	3	0.29	0.71	1.301	0.254		
		Deltamethrin	Alive	20	6	5	4	0.57	0.43	4.834	0.0279	50	R
			Dead	10	0	1	3	0.13	0.87	3.631	0.0567		
	Humera	DDT	Alive	27	14	6	4	0.71	0.29	3.685	0.0549	33	R
			Dead	10	0	2	5	0.14	0.86	1.041	0.308		
		Deltamethrin	Alive	24	7	6	5	0.56	0.44	2.935	0.0867	39	R
			Dead	9	2	2	4	0.38	0.62	1.646	0.2		
Afar	Amibara	DDT	Alive	30	6	6	13	0.36	0.64	5.617	0.0178	67	R
			Dead	10	0	1	4	0.1	0.9	2.531	0.112		
		Deltamethrin	Alive	24	3	3	8	0.33	0.68	6.529	0.0106	53	R
			Dead	9	0	0	5	0	1	–	–		
Gambela	Abobo	DDT	Alive	19	0	2	1	0.33	0.67	13.593	0.000227	37	R
			Dead	9	1	0	2	0.33	0.67	5.01	0.0252		
		Deltamethrin	Alive	30	11	7	3	0.73	0.27	3.827	0.05	33	R
			Dead	8	0	1	5	0.08	0.92	0.541	0.462		

## Discussion

The development of pervasive insecticide resistance across sub-Saharan Africa threatens to jeopardize the long-term effectiveness of both IRS and LLINs for malaria control [30, 31]. This study presents data from the largest nationwide, longitudinal monitoring of insecticide resistance among *An. arabiensis* populations to four classes of insecticides in Ethiopia. Intense resistance to pyrethroids (alpha-cypermethrin, deltamethrin, etofenprox, lambda-cyhalothrin and permethrin) and DDT were commonplace, and in many sites, vectors were able to survive exposure to five to ten times the diagnostic dose. These high levels of resistance are likely a direct consequence of historic DDT use for IRS, as well as its considerable application between 2000 and 2005, where 255,000–298,000 kg/year were used [32], alongside mass distributions of LLINs [2].

Patterns of resistance to bendiocarb, malathion, propoxur and pirimiphos-methyl also corresponded to shifts in the national insecticide policy [2]. Since 2012, *An. arabiensis* susceptibility to malathion increased in some areas, potentially attributable to the discontinuation of this insecticide for IRS; malathion was last used extensively for malaria control from 2003 to 2005 by the NMCP in areas with reported DDT resistance [5, 9]. Between 2011 and 2015, bendiocarb (with deltamethrin in 2011–2012) was the insecticide of choice for PMI-supported IRS activities in Oromia Region, where low levels of mosquito resistance were initially detected in 2012. However, because bendiocarb was largely abandoned due to its short residual efficacy, relative to other organophosphates and carbamates [33], only moderate levels of resistance developed in a few areas. Concurrent propoxur spraying in 2012 was accompanied by the emergence of potential resistance in some *An. arabiensis* populations by 2014 and likewise, decreased *An. arabiensis* susceptibility to pirimiphos-methyl was observed in 2016, concomitant with the switch to this insecticide in selected districts in 2015; however it should be noted that these sites fell outside the districts where pirimiphos-methyl was used for IRS. In general, the reactive, and in some cases, heterogeneous use of different insecticides has resulted in highly focal, volatile resistance profiles across sentinel sites [34], complicating the prospective deployment of interventions for vector control. Study observations are consistent with earlier cross-sectional evaluations from Ethiopia [35], which also describe widespread resistance to DDT and pyrethroids, as well as more restricted decreases in *An. arabiensis* susceptibility to malathion, bendiocarb and propoxur [5–15]. Furthermore, results from Sudan, Kenya and Eritrea corroborate large-scale resistance trends in *An. arabiensis* documented across this region [35–38].

Study results raised concerns pertaining to the comparability of WHO and CDC insecticide resistance tests. While in some areas, outcome measurements from both assays are reported to be equivalent in terms of resistance monitoring [39, 40], our data align with others reporting considerable discrepancies [28, 41, 42], which were problematic to interpret, particularly when susceptibility profiles annually fluctuated above or below the thresholds of resistance set by the WHO [18, 28]. Previous studies have suggested that the extent of inter-assay agreement may reflect levels of susceptibility heterogeneity, whereby tests conducted on vector populations with highly variable resistance profiles are more prone to inconsistent results [28]. Indeed, direct comparisons between our WHO and CDC bottle bioassays performed using bendiocarb and propoxur (where *An. arabiensis* were completely susceptible), and against pyrethroids (where resistance was more capricious) supports this supposition. Others have proposed that CDC bioassays may over-estimate pyrethroid resistance, as insecticide repellency can reduce the already relatively short contact time (30 min) of mosquitoes in coated bottles [40]. In this regard, our results demonstrated the opposite; higher and more uniform levels of pyrethroid resistance were obtained for WHO tests, when results were pooled across study sites.

There are a number of other technical and biological factors which could contribute to discordance between assays. WHO papers are distributed from a centralized source, which may render them prone to inter-batch variation and depending upon procurement schedules, can result in filter papers of different ages being used for the same monitoring activities in a given year. Ideally, to ensure consistency between study sites, all batches of papers would have been tested initially using a susceptible laboratory strain. CDC bottle bioassays are coated in-house which also introduces issues of standardization based on the proficiency of individual laboratory technicians, conditions of insecticide storage and numbers of consecutive times bottles are re-used [43–45]. *Anopheles* larvae were sampled from a range of the most productive breeding sites with different effective population sizes, genetic compositions, temperatures, nutritional access and chemical exposures depending upon local ecology. One important factor that was not investigated in this study was the influence that intensive agriculture pesticide use has had on resistance levels in Ethiopia (reviewed by [46]). Once collected, bioassays were conducted on emergent adult mosquitoes who were presumed to be *An. arabiensis*, based on PCR validation of a sub-set in 2015 and 2016. It should be noted that 10.4% of all PCR reactions did not amplify either because of technical errors or the specimens did not belong to the species under investigation (*An. gambiae* s.s., *An. arabiensis* or *An. quadriannulatus* species B/*An. amharicus*). Finally, WHO tests assess mortality after a 24-h holding period, while CDC bioassays measure knock-down and acute toxicity, which depending upon the degree of vector tolerance, may not necessarily be interchangeable [28].

Regarding the underlying mechanisms of resistance, the restoration of pyrethroid susceptibility following pre-exposure to PBO and the non-association of L1014F *kdr* allele frequency with levels of *An. arabiensis* mortality in WHO bioassays, suggests that both over-expression of detoxification enzymes and target-site mutations are driving insecticide resistance. Moderate to high *kdr* frequencies were detected in a number of sites and were fixed or approaching fixation in a minority. However, by comparison with earlier reports from the same areas, *kdr* allele frequencies were lower overall [9, 14, 47] and between our study years we also observed a slight, albeit not statistically significant, decline. In 2015, the lack of deviations from Hardy–Weinberg equilibrium in most areas indicated that selection for *kdr* heterozygotes was not ongoing, allowing this allele to be lost to genetic drift in some populations, but by 2016, study sites from five regions demonstrated evidence for *kdr* selection, potentially resulting from a mass distribution of LLINs by the NMCP beginning in August 2015. Given the lack of association between mosquito bioassay mortality and presence/absence of *kdr* mutation, there are likely to be other mechanisms at play in the development of resistance. Future local surveillance programmes may wish to consider screening for additional, recently identified genetic markers of metabolic resistance in *An. arabiensis*, e.g. *CYP6P4* [48].

## Conclusions

To date, the presence of insensitive acetylcholinesterase mutations (*ace*-*1* ^R^ or G119S), known to mediate resistance to both organophosphates and carbamates in *An. gambiae* s.s. and *An. arabiensis* [49, 50], has not been reported in Ethiopia among vectors resistant to malathion or propoxur [9]. The bioassay results in this study were not indicative of any cross-resistance both between organophosphates and carbamates and among chemicals belonging to the same insecticide class. This observation suggests the existence of additional metabolic resistance mechanisms that can confer insecticide-specific resistance and also has implications for the development of an insecticide resistance management strategy. If inter- and intra-class rotation of different insecticides could be exploited to reduce selection pressures, this may have the potential to safeguard continued efficacy of IRS and other vector control strategies in Ethiopia and proactively mitigate the development of future insecticide resistance. Furthermore, additional epidemiological studies are warranted, in parallel with future resistance monitoring activities, to determine the operational impact of insecticide resistance on malaria vector control in Ethiopia.

## Additional file

**Additional file 1: Tables S1.** Summary of PMI-supported IRS activities in Ethiopia, 2008-2016.** Table S2.** Summary of insecticide resistance assays conducted per year in Ethiopia, 2012-2016.** Table S3.** Percentage corrected mortality (and numbers tested) of *Anopheles arabiensis* in WHO susceptibility tests conducted in Ethiopia, 2013.** Table S4.** Percentage corrected mortality (and numbers tested) of *Anopheles arabiensis* in WHO susceptibility tests conducted in Ethiopia, 2014.** Table S5.** Percentage corrected mortality (and numbers tested) of *Anopheles arabiensis* in WHO susceptibility tests conducted in Ethiopia, 2015.** Table S6.** Percentage corrected mortality (and numbers tested) of *Anopheles arabiensis* in WHO susceptibility tests conducted in Ethiopia, 2016.** Table S7.** Percentage corrected mortality (and numbers tested) of *Anopheles arabiensis *in CDC bottle bioassays conducted in Ethiopia, 2013.** Table S8.** Percentage corrected mortality (and numbers tested) of *Anopheles arabiensis* in CDC bottle bioassays conducted in Ethiopia, 2014.** Table S9.** Percentage corrected mortality (and numbers tested) of *Anopheles arabiensis* in CDC bottle bioassays conducted in Ethiopia, 2015.** Table S10.** Percentage corrected mortality (and numbers tested) of *Anopheles arabiensis* in CDC bottle bioassays conducted in Ethiopia, 2016.** Table S11.** Percentage corrected mortality (and numbers tested) of *Anopheles arabiensis* in WHO susceptibility tests and CDC bottle bioassays conducted in Ziway Dugda, Ethiopia, 2013-2016.
